# Confinement-Engineered Superconductor to Correlated-Insulator Transition in a van der Waals Monolayer

**DOI:** 10.1021/acs.nanolett.1c03491

**Published:** 2022-02-15

**Authors:** Somesh Chandra Ganguli, Viliam Vaňo, Shawulienu Kezilebieke, Jose L. Lado, Peter Liljeroth

**Affiliations:** Department of Applied Physics, Aalto University, FI-00076 Aalto, Finland

**Keywords:** correlated insulator, superconductor, insulator to superconductor transition, monolayer niobium diselenide, scanning tunneling microscopy and spectroscopy

## Abstract

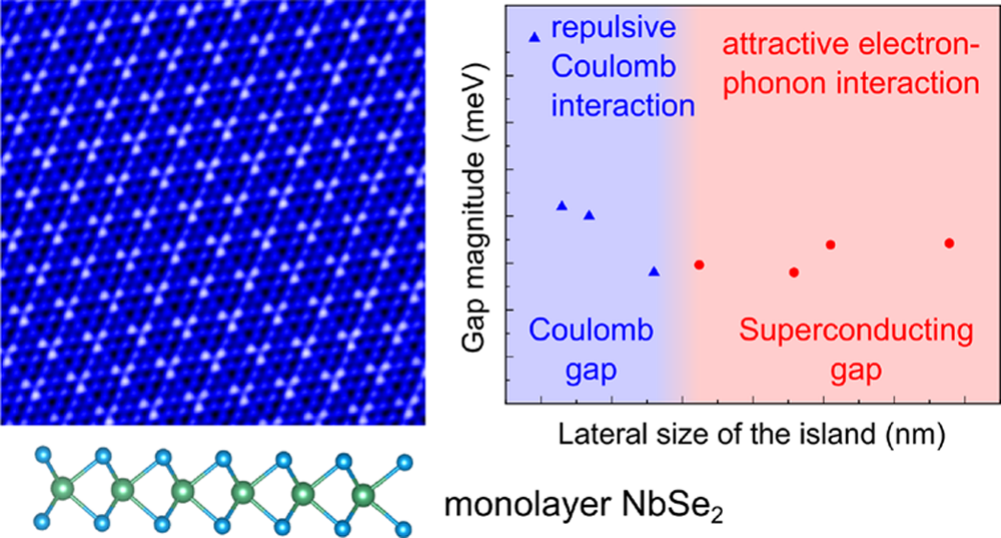

Transition metal dichalcogenides (TMDC) are a rich family of two-dimensional materials displaying a multitude of different quantum ground states. In particular, d^3^ TMDCs are paradigmatic materials hosting a variety of symmetry broken states, including charge density waves, superconductivity, and magnetism. Among this family, NbSe_2_ is one of the best-studied superconducting materials down to the monolayer limit. Despite its superconducting nature, a variety of results point toward strong electronic repulsions in NbSe_2_. Here, we control the strength of the interactions experimentally via quantum confinement and use low-temperature scanning tunneling microscopy (STM) and spectroscopy (STS) to demonstrate that NbSe_2_ is in close proximity to a correlated insulating state. This reveals the coexistence of competing interactions in NbSe_2_, creating a transition from a superconducting to an insulating quantum correlated state by confinement-controlled interactions. Our results demonstrate the dramatic role of interactions in NbSe_2_, establishing NbSe_2_ as a correlated superconductor with competing interactions.

Niobium dichalcogenides, and in particular NbSe_2_ is well-known to be a paradigmatic superconducting two-dimensional material, and it realizes Ising superconductivity at the monolayer (ML) limit.^1−3^ Because of its superconducting nature, NbSe_2_ has been considered to be a metal where Coulomb repulsions play a marginal role and the superconducting state arises from conventional electron–phonon coupling.^4^ Indeed, the emergence of charge density wave states is usually attributed to soft-phonon modes,^5−10^ so that symmetry broken states are not related with strong Coulomb interactions.

Despite the apparent marginal role of the Coulomb repulsion in NbSe_2_, related compounds in the dichalcogenide family show strong correlations.^11−15^ In particular, VSe_2_ is known to be a strongly correlated material^16^ with competing correlated states including a potential magnetic Mott insulating state.^14,17−19^ The chemical similarity between NbSe_2_ and VSe_2_, contrasted with their dramatically different electronic properties, motivates the question of whether NbSe_2_ exhibits a strongly correlated superconducting state, in contrast with the originally assumed weakly interacting scenario.^20−23^ In that regard, theoretical calculations have shown that NbSe_2_ is close to a Mott insulating transition to a ferromagnetic state.^20−23^ These results suggest that competing interactions coexist in NbSe_2_ system, and in particular suggest the possibility of the superconducting state coexisting with strong Coulomb interactions.

In this manuscript, we experimentally demonstrate that ML NbSe_2_ is in proximity to a correlated insulating state, by controlling the strength of the electronic interactions by quantum confinement effects. In particular, we show that for ML NbSe_2_ islands of size several times the coherence length, repulsive electronic interactions create a phase transition from a superconducting to a correlated insulating state. This behavior is rationalized from a competing interaction scenario (Figure 1a), in which attractive electron–phonon interactions compete with strongly repulsive Coulomb interactions. The electron–phonon interactions that give rise to a superconducting ground state do not depend on the system size and will dominate if the system size is increased sufficiently (Figure 1b). On the other hand, the repulsive Coulomb interactions are strongly dependent on the system size (*U* ∝ 1/*L*^24−26^) and will drive the system into a Coulomb-gapped, correlated state as the system size is decreased. This picture is complementary to the classical interpretation in terms of Coulomb blockade and completely analogous to the approach taken in, for example, interpreting the correlated insulating states in twisted bilayer graphene in terms of local repulsion.^26^ We test this behavior experimentally by tuning the size of NbSe_2_ islands and using low-temperature scanning tunneling microscopy (STM) and spectroscopy (STS) to measure the type and magnitude of the resulting energy gap. Our results provide a quantitative experimental bound on the strength of repulsive interactions of NbSe_2_, highlighting a nontrivial impact of correlations in superconducting dichalcogenides.

**Figure 1:**
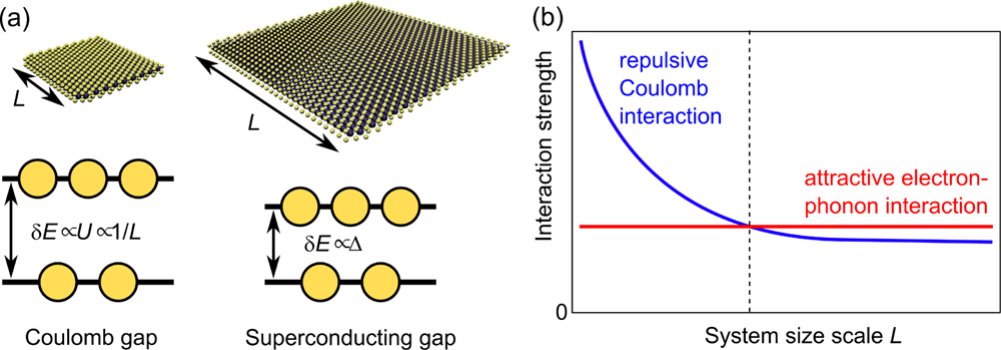
(a) Sketches of small and large NbSe_2_ islands with the associated Coulomb or superconducting gaps. (b) Schematic dependence of the attractive and repulsive interactions on the system size.

## Experimental Superconducting-Correlated Transition

**Figure 2:**
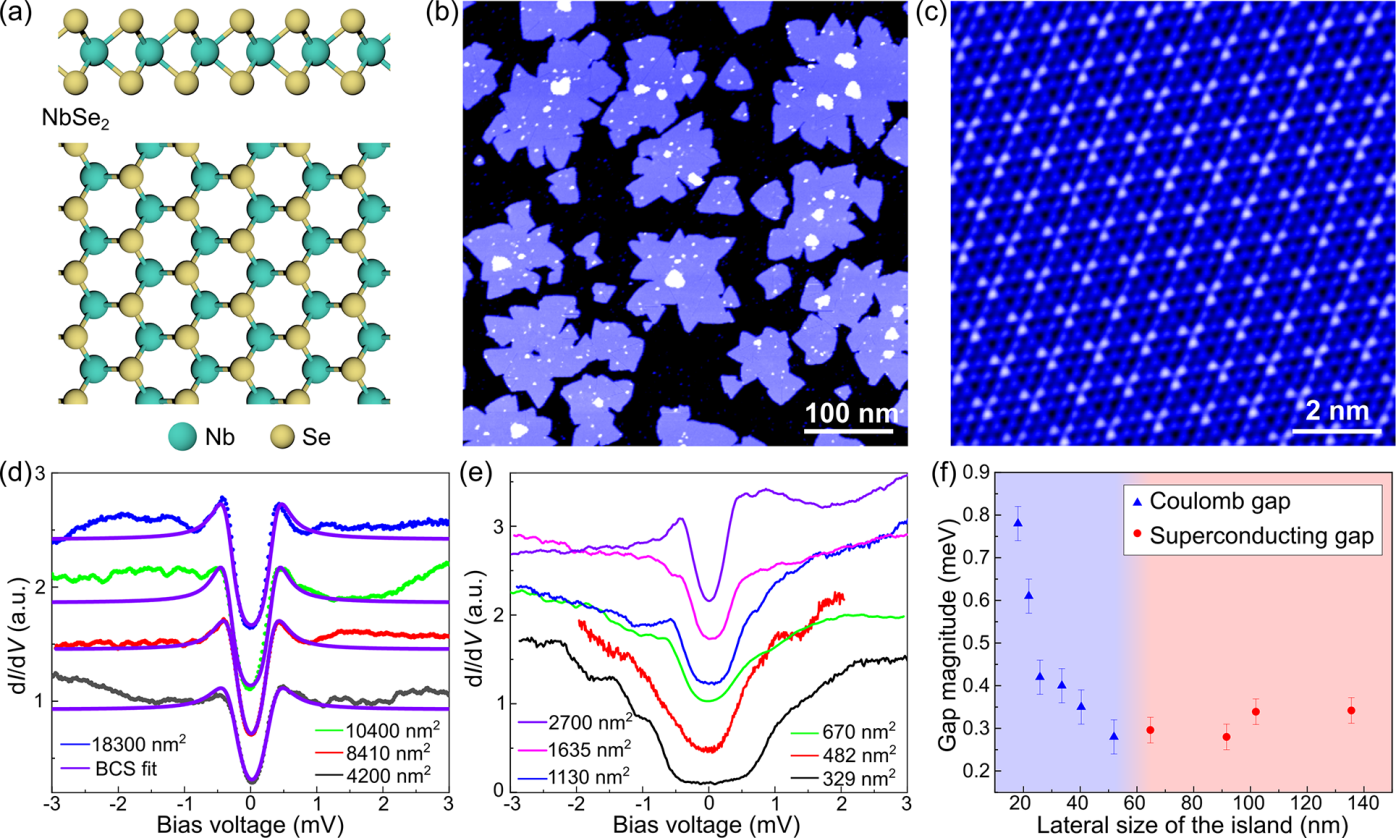
(a) Side and top view schematics of monolayer NbSe_2_. (b) Large-scale STM image of monolayer NbSe_2_ on HOPG showing a large variation of island sizes. (c) Atomic resolution image of monolayer NbSe_2_ showing 3 × 3 charge density wave modulation. (d,e) Variation of the superconducting (d) and Coulomb (e) gap with island size. Spectra have been normalized and offset vertically. Superconducting gaps in panel (d) have been fit with the Dynes equation (solid purple lines). (f) Evolution of the gap magnitude extracted from the tunneling spectra as a function of the island size showing a transition from Coulomb gap-like to superconducting spectra as the size is increased. The shape of the measured spectrum is indicated by the different symbols: blue triangles and red circles for the spectra exhibiting Coulomb- and SC-type gaps, respectively.

We grow NbSe_2_ (Figure 2a) on a highly oriented pyrolytic graphite (HOPG) substrate with a submonolayer coverage. By adjusting the growth conditions (see Supporting Information (SI) for details), we achieve a sample with a wide variety of island sizes and their relative separations. This creates an ideal platform to study the effects of quantum-confinement enhanced correlations. The island sizes vary between a few hundreds of nm^2^ to several tens of thousands of nm^2^ (lateral sizes few tens of nm to several hundreds of nm, see SI for island size determination). Figure 2b shows an STM image of a representative area (500 × 500 nm^2^), where this size variation of individual ML islands is apparent. Each individual island has atomically sharp edges and show the well-known 3 × 3 charge density wave (CDW) modulation similar to extended ML NbSe_2_ (Figure 2c). While the data shown in Figure 2c was acquired on a NbSe_2_ island with a lateral size of ∼92 nm (area 8400 nm^2^), the CDW modulation persists down to islands sizes of <500 nm^2^ (see SI). We characterize the electronic properties of each individual island by carrying out spatially resolved tunneling conductance (d*I*/d*V*) measurement (see Methods in SI for details). Typical examples of the d*I*/d*V* spectra are shown in Figure 2d,e. The spectra can be divided into two groups based on qualitative differences. Islands with sizes 4200 nm^2^ and above show density of states consistent with BCS-like behavior with particle-hole symmetric coherence peaks (Figure 2d), which indicate a presence of phase-coherent Cooper pairs. On the other hand, islands with sizes 2700 nm^2^ and below have distinctive particle-hole asymmetric density of states (Figure 2e) with no coherence peaks. This transition occurs at a size range several times larger than the coherence length of NbSe_2_ (∼7 nm, see below).

Such asymmetric differential conductance is typical of inelastic steps associated with correlated Coulomb excitations.^27−29^ Furthermore, the magnitude of the energy gap in these islands monotonically increases with decreasing island size (Figure 2f, the details of extracting the energy gap are given in the SI).^24,30^ This behavior is consistent with the presence of a Coulomb gap in small islands, where the repulsive Coulomb interaction dominates over phonon-mediated attractive interactions. On the other hand, the BCS-shaped superconducting gaps in the islands in Figure 2d are independent of the island size (Figure 2f) as the electron–phonon coupling strength does not depend on the system size. The Coulomb gap and superconducting gaps can also be distinguished by their respective magnetic field-dependent behavior (see SI).

Disorder driven superconductor–insulator transition (SIT) cannot justify our data, since we observe that the SC gap remains practically constant with reducing island size. The data also suggests that disorder does not have a sizable impact besides a small renormalization of the density of states as given by the Altshuler-Aronov effect.^31,32^ The main source of disorder appears to be on the edges of the islands which show slightly different SC energy gap compared to the center of the island (see SI).

## Theoretical Model for Competing Interactions

The previous phenomenology can be rationalized with a many-body low energy model. Many-body interactions are well-known to lead to Coulomb blockade effects in conventional superconductors, promoting intriguing phenomena arising from the interplay of pairing correlations and finite size effects.^33,34^ However, these phenomena have remained unexploited to probe many-body effects in correlated two-dimensional superconductors. Since the full quantum many-body system for a nanometer-sized island cannot be exactly solved, we will focus on the instability of the lowest energy 2*n* single-particle eigenstates of the NbSe_2_ island Ψ_*i*,*s*_ with *i* = 1,..., *n* as the state number and *s* = *↑*, *↓* as the spin quantum number. These states closest to the Fermi energy will be the ones most impacted by interactions, and therefore the fundamental physics of the system can be captured by projecting electronic interactions in this manifold. For the sake of concreteness, we take interactions *SU*(2) symmetric and constant on the Fermi surface manifold. In particular, we take projected electronic interactions partitioned into intraorbital repulsive ones *U* (of Coulomb origin) and interorbital attractive ones *V* (of electron–phonon origin). Furthermore, due to the existence of nearby large superconducting islands, the low energy states will feel a superconducting proximity effect with a value depending on the distance to the closest big superconducting island. We parametrize this effect with Δ̅. The half filling of the low energy manifold is enforced by μ, and computed self-consistently for each *U* and *V*. The low energy many-body Hamiltonian takes the form
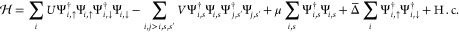
1

The projected electron–phonon interaction *V* is taken to be independent of the system size, whereas the projected Coulomb repulsive interaction *U* will get enhanced as the system size *L* becomes smaller as  due to the long-range tail of Coulomb interactions. The effective model is solved using exact diagonalization, projecting the electronic repulsion onto the lowest energy states and solving the projected Hamiltonian exactly. This is, of course, an approximate procedure when a finite number of states is considered, and we verified that our results are not qualitatively modified when including a higher number of orbitals. For such a many-body Hamiltonian the single-electron density of states can be computed as , where *E*_0_ is the many-body energy and |Ω⟩ the many-body ground state.

**Figure 3:**
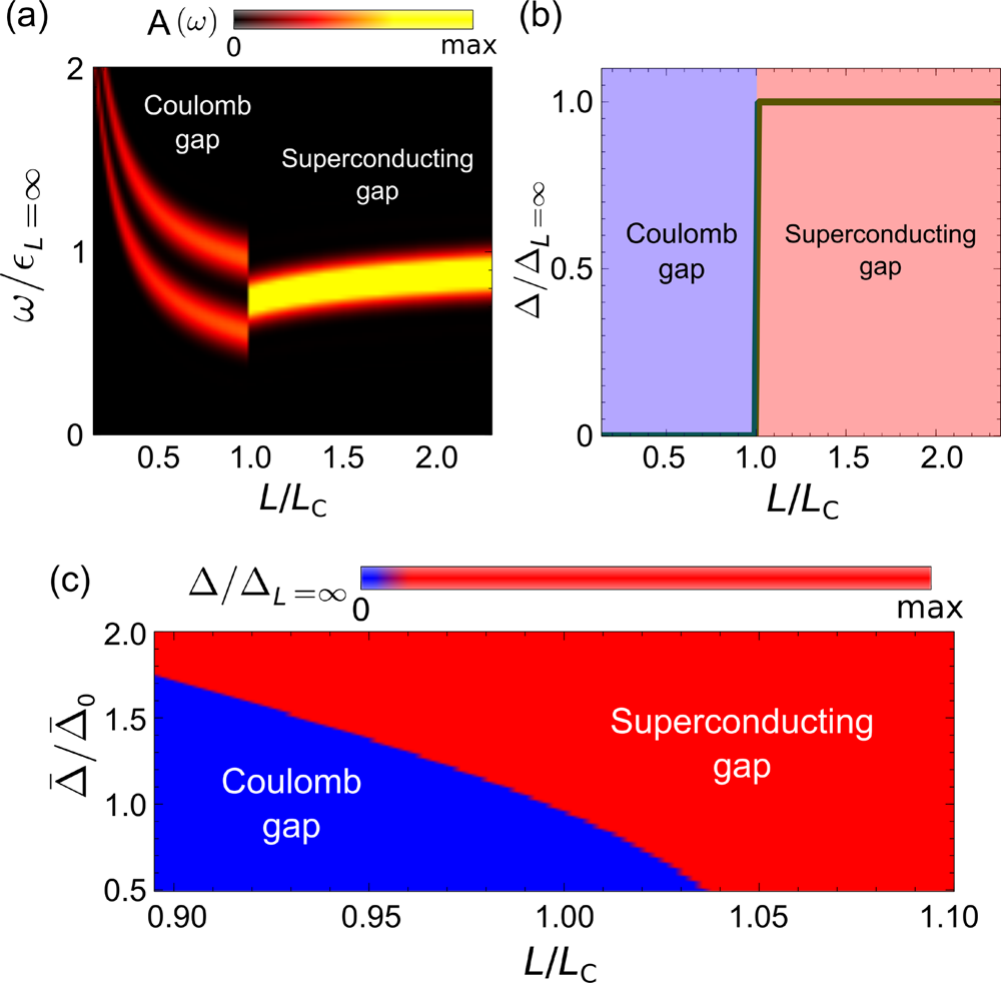
(a) Electron spectral function as a function of the system size and (b) induced superconductivity as a function of the system size. It is shown that a transition between the superconducting and the correlated state takes place without gap closing. For correlated islands close to the phase transition, increasing the superconducting proximity effect Δ̅ can push the system to the superconducting region as shown in panel (c). We took 2*n* = 8, *U*_0_ = 2 V and *L*_C_ is the critical length for Δ̅_0_ = 0.4 V.

We show in Figure 3a the single-electron spectral function *A*(ω) as a function of the system size *L*, where the transition between a Coulomb dominated gap ϵ_L_ to a superconducting dominated one can be seen. For large system size *L* → *∞*, the system shows a superconducting gap stemming from the attractive interactions and pinned by the superconducting proximity Δ̅. It is worth noting that, as the system is finite, observing a sharp phase transition from zero to finite superconducting order requires a finite value of the proximity effect Δ̅. Once the system size goes below a critical value *L*_C_, the nature of the excitation gap ϵ_L_ changes yet without a gap closing. The different nature of the two gaps above and below the transition point *L* = *L*_C_ can be verified by computing the superconducting expectation value Δ = *∑*_*i*_⟨Ψ_*i*,*↑*_Ψ_*i*,*↓*_⟩, showing that associated with the discontinuous jump as the size becomes smaller, the superconducting order parameter suddenly disappears (Figure 3b). We note that for small islands the observed spectra featuring a continuum of states above the gap are fundamentally different from the ones expected for systems with confined energy levels. The transition between the correlated gap for small islands and superconducting one for large islands is found to be of first order with a discontinuity on the gap. This is consistent with our experimental data and therefore strongly supports the competing interaction scenario.

Because of the proximity of NbSe_2_ to the phase transition point, it is expected that an external perturbation can cause a critical system to drift to different regions of the phase diagram. In particular, increasing a superconducting proximity effect Δ̅ would push the system toward the superconducting gapped region. This can be verified as shown in Figure 3c where it can be seen that ramping up the superconducting proximity pushes the system that originally has a correlated gap toward a superconducting gap. While this is shown for reduced range in Figure 3c, the very same mechanism applies in a broader range of *L* and Δ. We have verified that the same behavior remains qualitatively unchanged upon increasing the number of orbitals considered in the many-body Hamiltonian (shown in the SI).

It is well-known that 2H-NbSe_2_ exhibits charge-density wave order at low temperatures and the presence of Ising-type spin–orbit coupling might also have an effect on the observed behavior.^2,4,7,8,35^ However, by using a more detailed model incorporating these two effects (see SI), we can demonstrate that the observed phenomenology is a genuine Coulomb effect. Ising spin–orbit coupling leads to momentum dependent spin splitting in the Brillouin zone. As this perturbation respects time-reversal symmetry, it does not have a detrimental impact on spin-singlet superconducting state. Ising SOC will also not impact the Coulomb electronic interactions, as the momentum-dependent exchange splitting does not change the underlying atomic nature of the orbitals, which is the one that ultimately determines the strength of the local and nonlocal interactions.

The NbSe_2_ charge density wave gives rise to band folding and splitting, yet maintaining the system metallic. Even though the low energy states now become modulated in space following the CDW profile, the repulsive Coulomb interactions are not qualitatively affected. This is rationalized from the fact that electronic repulsion is an atomic property associated with the Wannier states, and thus again independent of larger scale structural reconstructions. The CDW also does not substantially impact the mechanism for superconductivity, because such reconstruction does not break time-reversal symmetry, the relevant symmetry that could strongly impact the spin-singlet superconducting state.

## Proximity Induced Quantum Phase Transition

**Figure 4:**
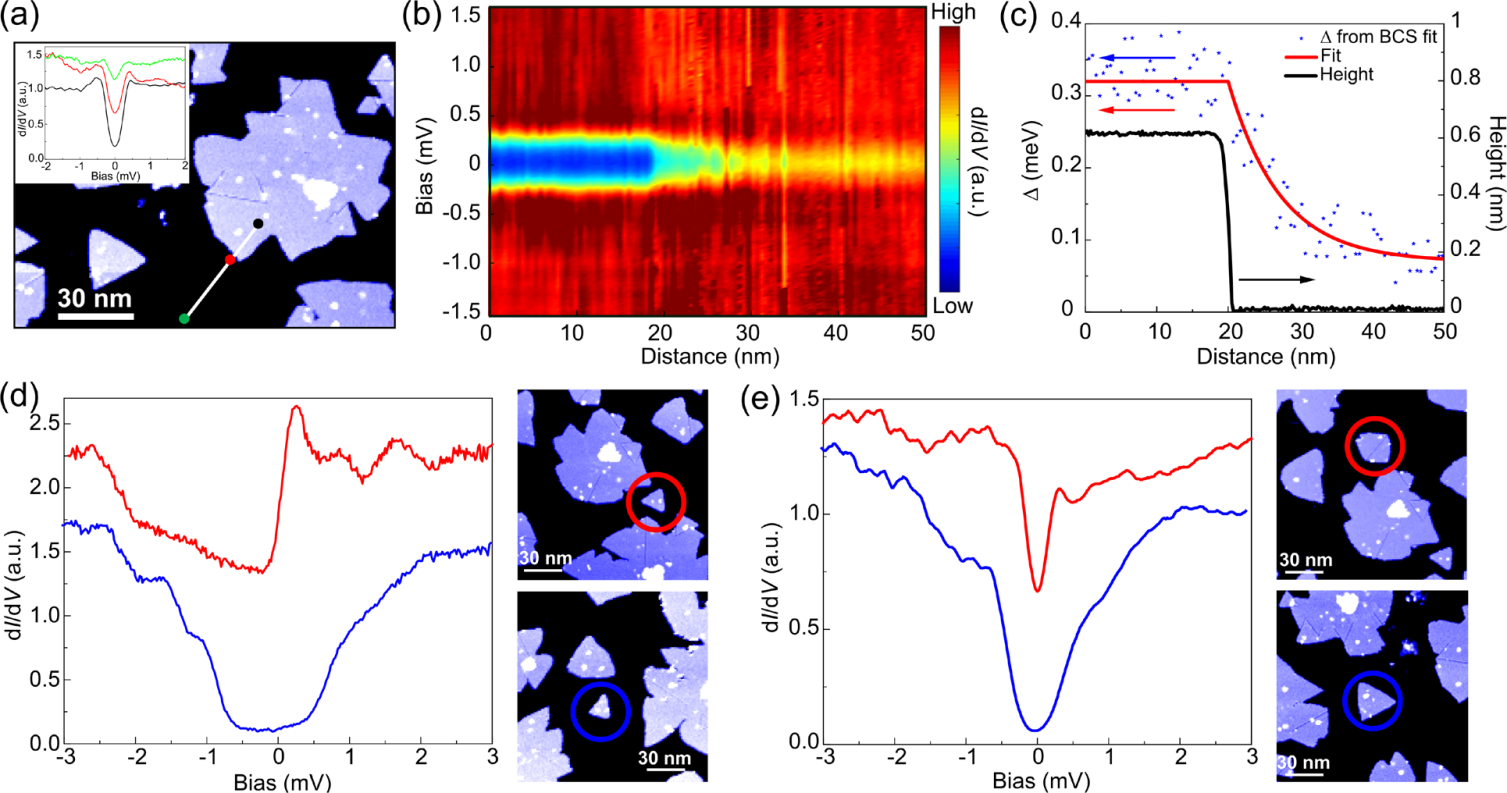
(a) STM image showing NbSe_2_ monolayer island. d*I*/d*V* spectra measured in black, green, and red points are shown in the inset with corresponding colors. (b) d*I*/d*V* spectra measured along the white line in panel (a) presented as a color scale plot. Black (green) point in (a) is the left (right) edge of (b). (c) Fitted SC gap, its exponential fit along with the height profile measured along the white line in panel (a). (d,e) Proximity induced superconductivity in Coulomb gapped islands. d*I*/d*V* spectra and topographic images of (d) an isolated island of size 330 nm^2^ (blue circle) and an island of size 330 nm^2^ in proximity with larger SC island (red circle), and (e) an isolated island of size 650 nm^2^ (blue circle) and an island of size 650 nm^2^ in proximity with larger SC island (red circle). Scale bars, 30 nm. Spectra in panels (d) and (e) are offset vertically for clarity.

On the basis of the previous results, we check this proximity-induced phase transition experimentally by comparing the spectra of different critical islands with different respective distances to a big superconducting island, probing whether the superconducting proximity effect transforms the correlated gap into a superconducting one. We start by quantifying the proximity effect in the NbSe_2_/HOPG-system as shown in Figure 4a–c. Measuring d*I*/d*V* spectra close to a SC NbSe_2_ shows a proximity-induced gap on HOPG and tracking the spatial evolution allows us to estimate the decay length. Fitting the spatially dependent d*I*/d*V* in Figure 4b to Dynes equation, we extract the gap as a function of the distance from the NbSe_2_ island edge (Figure 4c). An exponential fitting of Dynes gap with distance yields ξ ≈ 7 nm (see SI).

We then proceed to show the effect of proximity in the nonsuperconducting islands showing size-dependent Coulomb gaps. We selected two representative island sizes of 330 and 650 nm^2^ (Figure 4d,e, additional results on spatially resolved spectroscopy are shown in the SI). Here, the smaller of the islands is well into the Coulomb gapped regime, but the larger one is closer to phase transition determined in Figure 2f. When each of these islands are not in proximity (∼7 nm) to any superconducting islands (Figure 4d,e), they show particle-hole asymmetric Coulomb gap (Figure 4d,e, blue lines). Island with size 650 nm^2^ in proximity to a larger superconducting island shows a drastically different conductance with gap value comparable to the BCS gap observed in larger islands (Figure 4e, red line), indicating that the proximity effect is sufficient to push the system into the superconducting phase. Strong particle-hole asymmetric feature indicates significant presence of correlation in this proximity-induced superconducting island. The magnetic field-dependent behavior of proximitized island is also indicative of the presence of superconducting order (see SI). On the other hand, an island with a size of 330 nm^2^ in proximity to a larger superconducting island shows a complex spectra with no clear gap signature (Figure 4d, red line), indicating that the proximity-induced Josephson coupling is not sufficient to overcome Coulomb repulsion to induce superconducting order in this island.

## Conclusions

We have demonstrated that ML NbSe_2_ can be pushed to a correlated regime, driving a quantum phase transition from superconducting to a correlated gap. This transition is rationalized from the existence of competing interactions, in which the coexistence of attractive electron–phonon interactions, driving superconductivity, and repulsive Coulomb interactions, driving correlated insulating behavior, allows to dramatically change the nature of the ground state in NbSe_2_ by slightly enhancing the Coulomb interactions. The Coulomb gap observed in our system is inherently different from the single-particle gap observed in small metallic islands.^36^ The d*I*/d*V* spectra in the smallest NbSe_2_ islands (Figure 2e) show a continuum of states above the gap rather than a discrete set of states,^37^ indicating many-body nature of the gap in our system. While it is possible to analyze our data using the Coulomb blockade model typically employed for 3D superconductors,^38−40^ it is worthwhile to note that these systems are weakly interacting being far from any Stoner instability and electron induced symmetry breaking, whereas NbSe_2_ is in close proximity to correlated state which can be driven by perturbations such as strain.^23^ Also, the SIT mechanism observed here veers away from the traditional disorder-driven scenario. In comparison, similar SIT has been observed by controlling electronic interactions in twisted van der Waals multilayers.^41^

The critical role of Coulomb interactions highlighted in our results suggests a potentially crucial impact of electronic correlations for the emergence of both charge density wave orders and superconductivity besides the typical electron–phonon driven scenarios. Recent results show the presence of spin-fluctuations in ML NbSe_2_^42^ and nematic superconductivity in few layer NbSe_2_,^43^ which are indicative of its proximity to correlated regime. We finally showed that for correlated NbSe_2_ samples close to the phase transition, superconducting proximity effect strongly impacts the ground state, pushing the system through the superconductor-correlated phase boundary. Ultimately, these results suggest that due to the close to critical behavior of NbSe_2_, correlated states could be promoted in NbSe_2_ by screening,^41^ chemical,^44^ or twist engineering,^45^ putting forward *d*^3^ chalcogenides as paradigmatic strongly correlated two-dimensional materials.

## Supporting Information Available

Experimental methods, and additional experimental and theoretical results (PDF)
