# Supplementary material for: Confinement-Engineered Superconductor to Correlated-Insulator Transition in a van der Waals Monolayer
Source: Nano Lett. 2022 Feb 15;22(5):1845–50. doi: 10.1021/acs.nanolett.1c03491 (PMC8915256; doi:10.1021/acs.nanolett.1c03491)
Supplement: Supplementary file 1 — nl1c03491_si_001.pdf [file nl1c03491_si_001.pdf]

Supporting Information: Confinement-engineered superconductor to correlated-insulator transition in a van der Waals monolayer

Somesh Chandra Ganguli,* Viliam Vaňo, Shawulienu Kezilebieke, Jose L. Lado,* and Peter Liljeroth*

Department of Applied Physics, Aalto University, FI-00076 Aalto, Finland

E-mail: somesh.ganguli@aalto.fi; jose.lado@aalto.fi; peter.liljeroth@aalto.fi

Methods

MBE growth. Sub-monolayer $NbSe_2$ was grown by molecular beam epitaxy (MBE) on highly oriented pyrolytic graphite (HOPG) under ultra-high vacuum conditions (UHV, base pressure $\sim 1 \times 10^{-10}$ mbar). HOPG crystal was cleaved and subsequently out-gassed at $\sim$ 400°C. High-purity Nb and Se were evaporated from an electron-beam evaporator and a dual-filament low temperature Knudsen cell, respectively. The flux ratio of Nb to Se was controlled to be $\sim 1 : 30$. During the growth the substrate temperature was kept at $\sim$ 330°C, and after the growth the sample was annealed at the same temperature for 1 hour. The growth speed was determined by checking the coverage of the as-grown samples by scanning tunneling microscopy (STM). For higher temperature growth ($>$ 500°C), we obtain significant proportion of 1T-$NbSe_2$ islands.

STM/STS measurements. Subsequent to the growth, the sample was transferred to a low-temperature STM (Unisoku USM-1300) housed in the same UHV system. STM imaging and STS experiments were performed at $T = 350$ mK. STM imaging was performed in constant current mode. Differential conductance ($\mathrm{d}I/\mathrm{d}V$) spectra were measured using standard lock-in techniques sweeping the sample bias in an open feedback loop with rms bias modulation of 70 μV at a frequency of 873.7 Hz.

Determination of the island size. The size of the islands were determined using WSxM software,[S1] where the area of the feature size having height $\sim$ 6 Å (monolayer height) was used as the area of a monolayer island (Fig. S1). Lateral size was determined as $\sqrt{area}$.

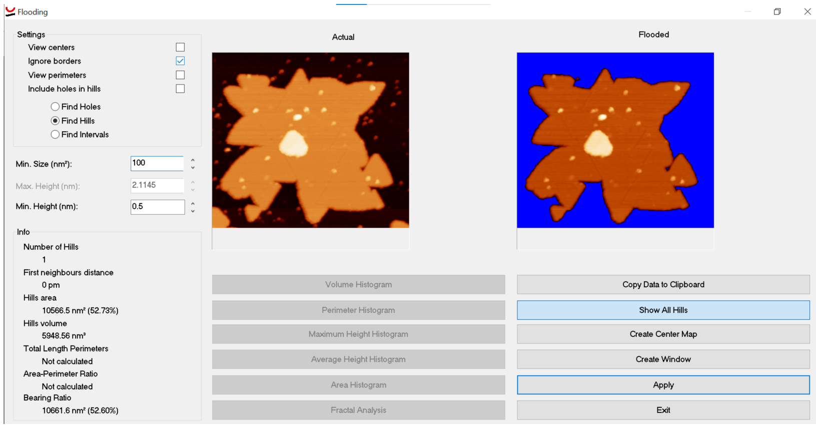

Figure S1: Determination of monolayer $NbSe_2$ island size using WSxM software.

Determination of the SC and Coulomb gap magnitudes. For determining the average SC gap, spectra were averaged over a 12.5×12.5 nm^2 area 10 nm away from the edge of the island. The data in Fig. 2 was recorded on islands that were non-proximitized (i.e. had no other islands in their immediate vicinity).

At first, the $\mathrm{d}I/\mathrm{d}V$ spectra on the superconducting islands were normalized with respect to the normal state conductance. These normalized $\mathrm{d}I/\mathrm{d}V$ spectra had a temperature independent V-shaped background at above superconducting T_c which was also apparent in the lowest temperature (at 350 mK) spectra as shown in Fig. S2(a). This is the well known Altshuler-Aronov effect due to electronic interactions. So, this background was subtracted

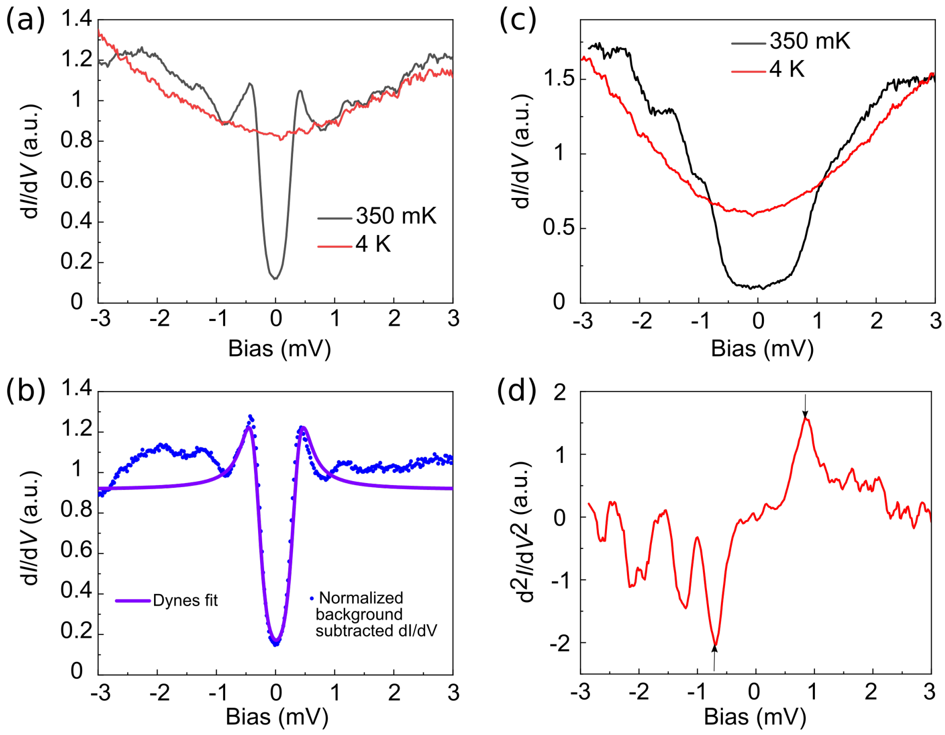

Figure S2: Background removal and fit of SC spectra and estimation of Coulomb gap: (a) Normalized 350 mK and 4 K spectra, taken in 18400 nm^2 island. (b) 350 mK spectrum after removing the 4 K background along with its Dynes fit. (c) Coulomb gap spectra taken in 329 nm^2 island (spectrum at $T = 4$ K shown for reference. (d) Numerical derivative d$^2I/$dV^2 of the 350 mK spectrum (arrows indicate Coulomb gap energies).

by dividing the superconducting spectra with the 4K spectra and the resultant spectra was fitted to the Dynes function $N_S(E) = \Re(\frac{|E|+i\Gamma(T)}{\sqrt{(|E|+i\Gamma(T))^2-\Delta(T)^2}})$ as shown in Fig. S2(b). Here, $N_S(E)$ denotes the normalized background subtracted SC density of states, $\Re$ denotes the real part, $\Gamma(T)$ is the temperature dependent quasiparticle lifetime broadening parameter, also known as the Dynes parameter and $\Delta(T)$ is the temperature dependent SC energy gap. The size dependence of the extracted Dynes parameter is shown in Fig. S3.

To extract the Coulomb gap magnitude the raw spectra (Fig. S2(c)) was numerically differentiated to obtain d$^2I/$dV^2, which has a characteristic peak-dip features in positive and negative biases respectively, as shown by arrows in Fig. S2(d). The average bias voltage location for peak and dip was taken to be the Coulomb gap.

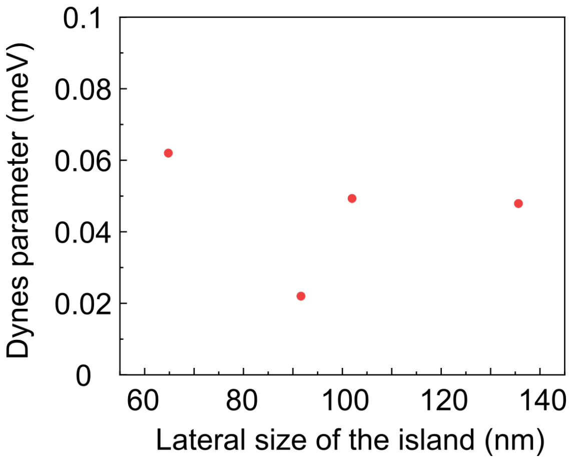

Figure S3: Size dependence of Dynes parameter from the fit with respect to the size of the SC islands.

Size dependence of CDW and band structure

In Fig. S4(a,c,e), we show the presence of 3×3 charge density wave in islands of sizes 480 nm^2, 1130 nm^2 and 9500 nm^2 respectively as indicated by their respective fast Fourier transforms (FFT) in Fig. S4(b,d,f). This indicates that the 3×3 CDW modulation present in the extended monolayer $NbSe_2$ survives down to the length scales where correlations are observed. Also, the intensity of the CDW modulation becomes inhomogeneous in the correlated regime. This is a further signature of the strengthening of interactions; perhaps such a change could be associated to an additional charge ordering promoted by interactions. In Fig. S4(g), the large bias $\mathrm{d}I/\mathrm{d}V$ remains unchanged with island sizes showing the characteristic Nb d-band feature observed in the extended monolayer. All these results clearly demonstrates that the 2H-polytype of $NbSe_2$ survives down to the length scales where correlations are being observed.

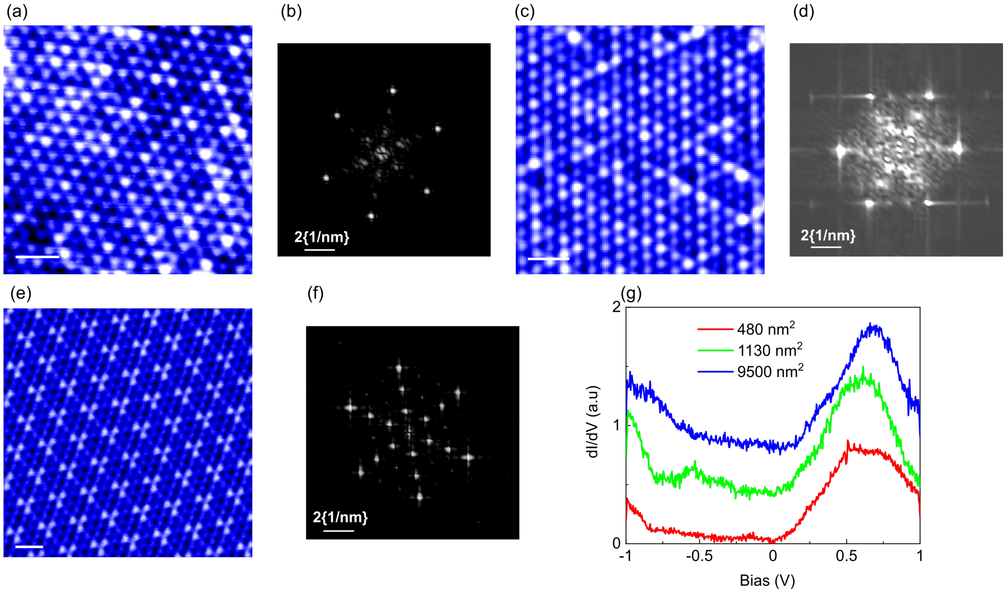

Figure S4: Size dependence of CDW and band structure. Examples of STM images and the corresponding FFTs showing the presence of the CDW state for $NbSe_2$ islands of different sizes: (a),(b) Island size 480 nm^2, (c),(d) Island size 1130 nm^2, (e),(f) Island size 9500 nm^2. Scale bars 1 nm. (g) Large bias range dI/dV spectra showing the presence of the Nb d-band at the Fermi level for all island sizes.

Magnetic field dependence

In Figure S5, we characterise and distinguish the different types of observed spectra by their respective magnetic field dependencies. The representative spectra chosen are superconducting (SC) spectra from island of size 4200 nm^2 (Fig. S5(a)), Coulomb gapped spectra from island of size 650 nm^2 (Fig. S5(b)), and proximitized SC spectra from island of size 650 nm^2 (Fig. S5(c)). The Coulomb nature of the island having size 2700 nm^2 becomes evident from the dI/dV taken at magnetic field of 3 T, which is greater than H_{c2} for the superconducting islands. As shown in Fig. S5(d), the superconducting island having size close to the SC-Coulomb phase boundary (4200 nm^2) and larger SC island (8400 nm^2) have almost indistinguishable dI/dV at $H = 3$ T whereas in the 2700 nm^2 island, significantly prominent gap signature is present easily distinguishable from the larger SC islands. The fitted BCS gap magnitude for island of size 4200 nm^2 decreases monotonically to zero with increasing field, whereas Coulomb gap in 650 nm^2 island remains finite and almost constant

(Fig. S5(e)). The normalized zero bias conductance (ZBC) of both SC and proximitized SC spectra monotonically increases to one with increasing field whereas ZBC for Coulomb gap remains almost constant with increasing field (Fig. S5(f)).

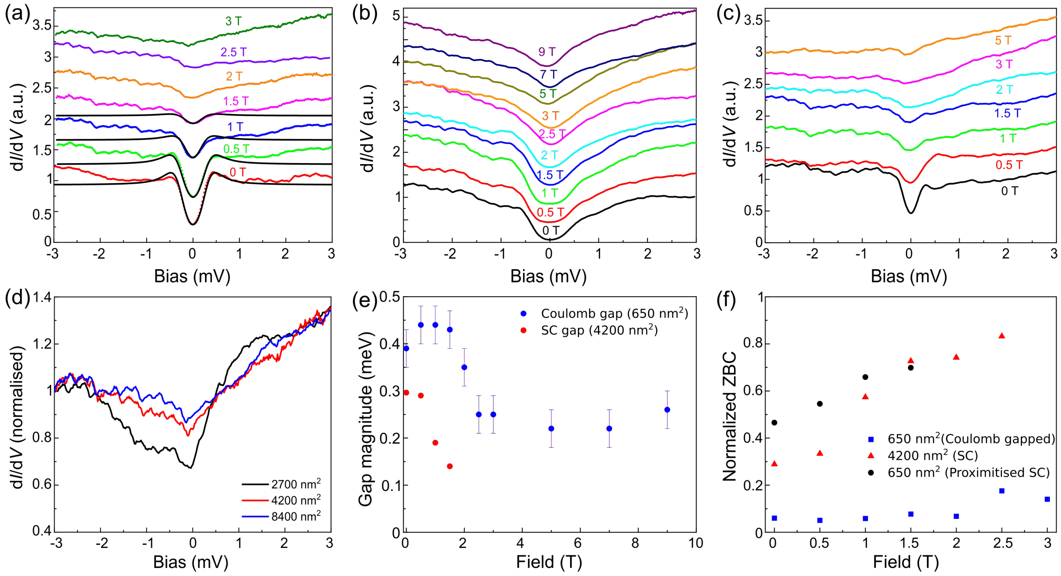

Figure S5: Magnetic field dependence of SC gap, Coulomb gap and proximitised SC gap. Magnetic field dependence of dI/dV spectra in island of size (a) 4200 nm^2 including BCS fits up to 1.5 T, (b) 650 nm^2, (c) 650 nm^2 having proximity to SC island. (d) Comparison of dI/dV taken at 3 T on islands with sizes of 2700, 4200 and 8400 nm^2. (e), (f) Magnetic field dependence of (e) gap magnitude and (f) normalized zero bias conductance. Spectra in panels (a),(b),(c) are offset vertically for clarity.

It is worthwhile to note that even for large SC islands we still have a small residual gap at high magnetic fields which could be attributed to a correlated pseudogap state that appears when the superconductivity is quenched, and can have additional symmetry broken states of charge or spin-ordering. Precise nature of this pseudogap state remains an open question in the field of correlated superconductors. It is noted that the small residual gap in high fields observed in Pb islands was attributed to the Coulomb gap.[S2]

Spatial dependence of point spectra within a single island

In Fig. S6(a,b), we demonstrate the representative spectra in edge and middle of a Coulomb-gapped island indicating that the magnitude of the Coulomb gap remains constant. Spatial variation of SC spectra is demonstrated in Fig. S6(d,g) for 2 different sized islands in Fig. S6(c,f) having areas 10400 nm^2 and 18400 nm^2, respectively (lateral sizes 102 nm and 136 nm, respectively). It is observed that the edges of the SC islands shows larger SC gap compared to the middle of the islands and this variation is larger for bigger islands. In fig. S6(e), the linespectra across CDW domain wall in fig. S6(c) (shown in red dotted line), indicates unchanged SC gap. Fig. S6(h) shows the spatial variation of the fitted SC gap with Dynes equation for spectra taken in island in Fig. S6(e). Fig. S6(i) shows spatial asymmetry: $(\mathrm{d}I/\mathrm{d}V(V = V_{\mathrm{cp}}) - \mathrm{d}I/\mathrm{d}V(V = -V_{\mathrm{cp}})$, where V_{cp} is the bias corresponding to the coherence peak positions).

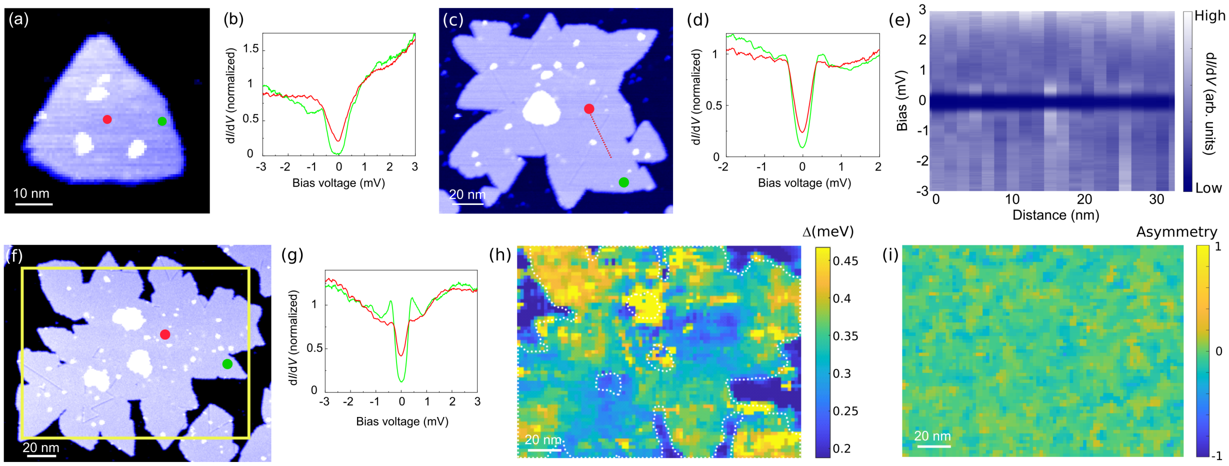

Figure S6: Spatial variation of dI/dV on island sizes. (a) Island size 650 nm^2. (b) Point spectra taken at indicated locations in (a). (c) Island size 10400 nm^2. (d) Point spectra taken at indicated locations in (c). (e) Line spectra across the domain wall indicated by the red dotted line in (c). (f) Island size 18400 nm^2. (g) Point spectra taken at indicated locations in (f). (h) Superconducting gap map from the Dynes fit of the dI/dV map taken at the area indicated by yellow rectangle at (f). The outline of the island's topographic feature is indicated by white dashed line. (i) Spatial asymmetry map.

Superconducting gap map and zero bias conductance map of smallest superconducting island

For the smallest island on which the superconducting spectra was observed (size $\sim$ 4200 nm^2), dI/dV map was obtained over an area 12.5 nm $\times$ 12.5 nm (Fig. S7(a)). The gap obtained from the fitted spectra with Dynes model and the normalised zero bias conductance shows spatial variation as seen in Fig. S7(b,c). It is also apparent from the spatial variations that the regions where ZBC is higher, SC gap is lower and vice versa. ZBC vs SC gap plot fit gives a slope of $\sim$ -0.54 indicating strong anticorrelation.

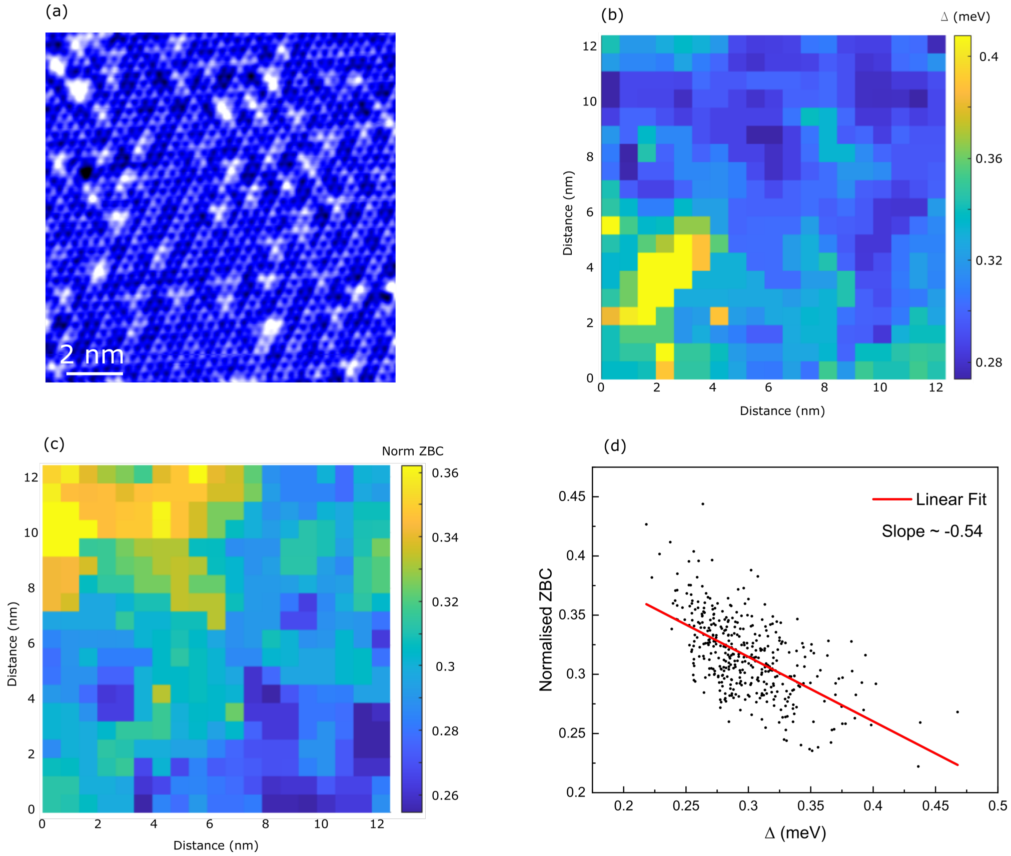

Figure S7: Spatial variation of superconducting gap and ZBC in superconducting island of size 4200 nm^2. (a) Atomic resolution of the area. (b) Spatial variation of fitted SC gap. (c) Spatial variation of ZBC. (d) ZBC vs SC gap along with its linear fit yields a slope of -0.54.

Dependence of the transition on the number of many-body orbitals

Here we show that the transition between the correlated gap and the superconducting gap takes place independently on the number of orbitals considered in the calculation. In particular, we show in Fig. S8 the spectral function as a function of the size of the island, taking a different number of many-body orbitals in the calculations. It can be clearly seen that both for $2n = 10$ (Fig. S8(a)) and $2n = 12$ (Fig. S8(b)) orbitals, a transition between a correlated gap to a superconducting one emerges, analogous to the calculations of Fig. 3(a) in the main manuscript. In the absence of U or V there would not be a phase transition between the correlated and superconducting regimes. Nevertheless, in the absence of repulsive interactions, there could still be a phase transition as function of the system size between a single-particle gap coming from quantized energy levels and a superconducting gap. This transition would have an associated smooth evolution of the gap, in contrast with the sharp transition we observe in our data. We finally note that the zero-bias anomaly can be modelled with the model of the dynamical Coulomb blockade.[S3] While modelling the dynamical Coulomb blockade in this system could be very interesting, it is significantly beyond the scope of this manuscript.

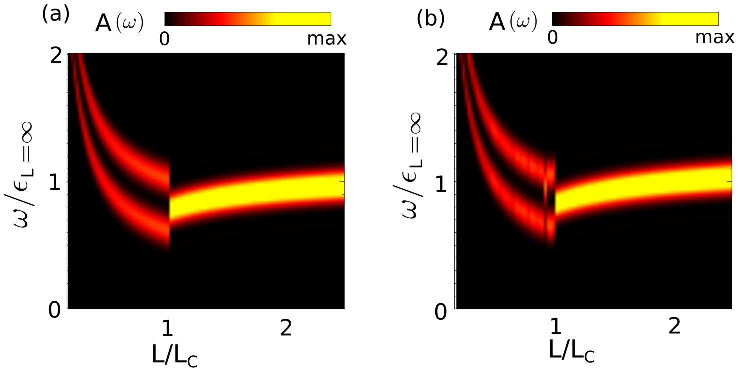

Figure S8: Electronic spectral function as a function of the size of the island, for a different number of many-body orbitals, $2n = 10$ in (a) and $2n = 12$ in (b). It is observed that the transition between different gaps happens irrespective of the number of orbitals.

SC-Coulomb phase boundary as a function of the strength of the proximity effect

Here we address the dependence of the transition between the correlated and superconducting state driven by proximity. First, it is worth noting that in the absence of superconducting proximity, the $NbSe_2$ would not show the presence of superconductivity due to its finite nature. However, for intermediate islands, the existence of a small proximity drives the system to the superconducting state. The critical length at which such transition takes place depends on the strength of the proximity effect as shown in Fig. S9. The transition remains sharp for finite proximity effects, illustrating that a sharp transition with system size is expected irrespective of the exact value of the superconducting proximity effect.

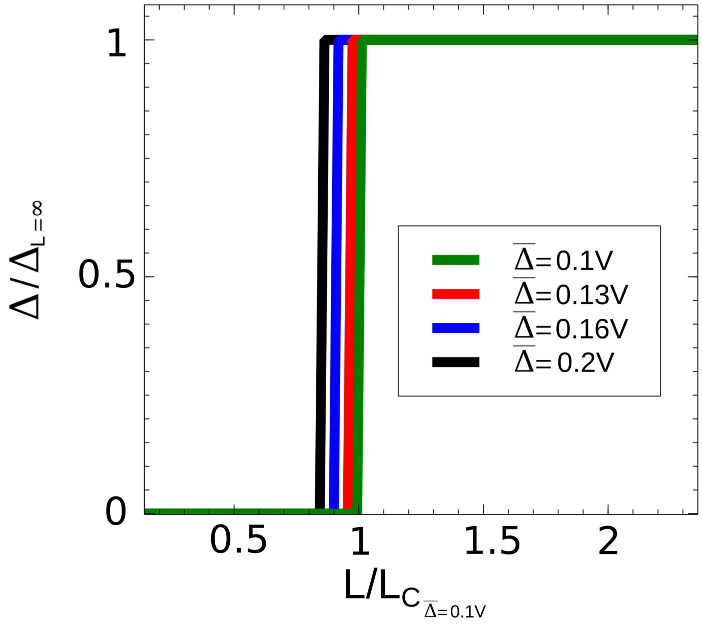

Figure S9: Transition from a correlated gap to a superconducting gap as a function of the size of the island for increasing values of the superconducting proximity effect.

Coulomb-enhanced interactions including charge-density wave and spin-orbit coupling

$NbSe_2$ is well known for having a complex electronic structure combining Ising spin-orbit coupling and charge density wave. In that regard, it is worth considering whether those effects would have a non-trivial interplay with the confinement enhanced interaction. In this section, we address this issue, showing that Ising spin-orbit coupling nor charge density wave qualitatively change the picture presented in the main manuscript.

In the following we will consider an atomistic tight binding model for the Wannier states of the $NbSe_2$ band, one per Nb atom, sitting in a triangular lattice.[S4] The total Hamiltonian takes the form

$$H = H_{\text{kin}} + H_{\text{CDW}} + H_{\text{SOC}} \tag{1}$$

where H_{kin} is the spin-independent hopping term

$$H_{\text{kin}} = \sum_{i,j,s} t_{ij} c^{\dagger}_{i,s} c_{i,s} \tag{2}$$

H_{CDW} is the charge density-wave order

$$H_{\text{CDW}} = \sum_{i,s} \epsilon_{\text{CDW},i} c^{\dagger}_{i,s} c_{i,s} \tag{3}$$

and H_{SOC} is the intrinsic Ising spin-orbit coupling

$$H_{\text{SOC}} = i\lambda_{\text{SOC}} \sum_{\langle ij \rangle,s,s'} \gamma_{ij} \sigma_z^{s,s'} c^{\dagger}_{i,s} c_{i,s'} \tag{4}$$

In the previous terms, t_{ij} is the hopping term that incorporates up to 4th-neighbor hopping, $\epsilon_{\text{CDW},i}$ are the modulated onsite energies associated to the CDW order, $\nu_{ij} = \pm 1$ alternate signs between the different bonds leading to a C_3 symmetric hopping,[S5] and σ_z is

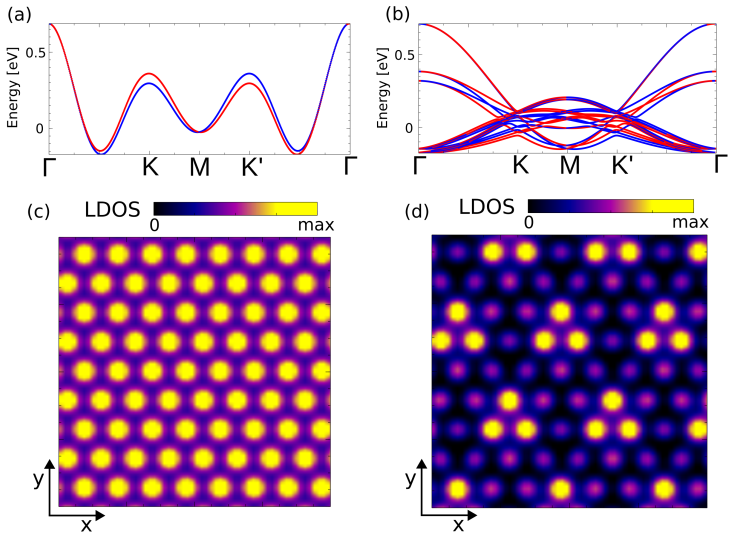

Figure S10: Electronic structure of $NbSe_2$. Band structure for the minimal unit cell in the absence of CDW (a), and LDOS (c). Panel (b) shows the band structure in the presence of CDW for a 3x3 supercell, and panel (d) shows the LDOS reflecting the spatial charge modulation of the CDW.

the spin Pauli matrix. The band structure and local density of states at Fermi energy are shown in Fig. S10. It is observed that the bands feature the momentum dependent spin-splitting associated to the Ising spin-orbit coupling, and that in the presence of the CDW perturbation the local density of states (LDOS) reproduces the experimentally observed features. As a result, the previous model captures all the microscopic features of $NbSe_2$.

With the previous Hamiltonian, we now consider the effect of interactions on a finite island. We include interactions in the form of long-range Coulomb interaction in the atomistic model as

$$H_{\text{Coulomb}} = \sum_{i \neq j, s, s'} \frac{V_0}{|\mathbf{r_i} - \mathbf{r_j}|} c^\dagger_{i,s} c_{i,s} c^\dagger_{j,s'} c_{j,s'} \tag{5}$$

where $\mathbf{r_i}$ is the location of Nb atom i, and V_0 is the Coulomb prefactor in atomic units. As this term turns the system into a full-fledge many-body problem, we will consider the impact of interactions only in the lowest energy states. We project this Coulomb interaction to the states closest to the Fermi surface Ψ_α, giving rise to an interaction of the form

$$\mathcal{H}_{\text{Coulomb}} = \sum_{ijkl} \mathcal{V}_{ijkl} \Psi_i^\dagger \Psi_j \Psi_k^\dagger \Psi_l \tag{6}$$

where $\mathcal{V}_{ijkl}$ are obtained by projecting Eq. 5 into the low energy states Ψ_α. We now take finite-size islands with different shapes and different number of atoms, and compute the effective interaction $\mathcal{V}_{ijkl}$. We perform this procedure on islands whose atomistic Hamiltonian has zero and non-zero spin-orbit coupling and zero and non-zero charge density wave, the results are shown in Fig. S11. As the effective interaction $\mathcal{V}_{ijkl}$ is a four dimensional tensor, generically complex-valued, we will characterize the strength of the repulsive interactions by the average of its absolute value. As shown in Fig. S11, we observe a robust $1/L$ behavior of the projected interaction, independently on the presence or absence of Ising spin-orbit coupling and charge density wave. These results demonstrate that not Ising spin-orbit cou-

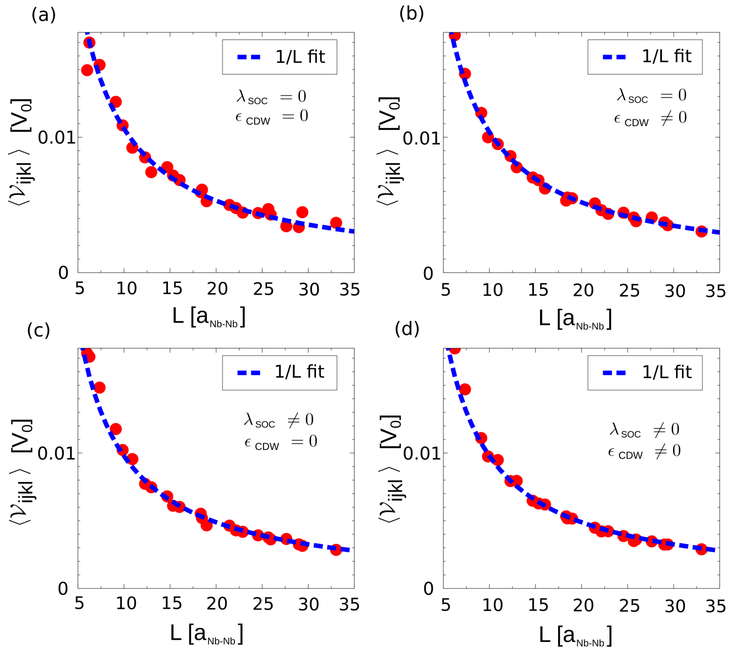

Figure S11: Confinement controlled Coulomb interactions. Average value of the projected interaction $\mathcal{V}_{ijkl}$ on the Fermi surface states Ψ_α. The projected interaction is computed for model zero SOC and zero CDW (a), zero SOC and finite CDW (b), zero CDW and finite SOC (c), and finite CDW and finite SOC (d) It is observed that the average repulsive interaction shows a $1/L$ dependence, with L the size of the island, in agreement the model in the main manuscript.

pling nor the charge density wave create an impact on the projected interactions and that the repulsive interactions are purely dominated by the island confinement effect.

We now comment on the possibility of extracting the exact parameters for our theoretical model from the experimental data. Extracting U_0 and c_0 and the full dependence of L_{C} on U_0, c_0, V, μ, and $\bar{\Delta}$ would require a very large number of islands so that a detailed error analysis can be done when performing the fitting. Otherwise, small fluctuations would give rise to an inaccurate estimate of U_0 and c_0. Since at the current stage, our experiments do not allow for a precise estimate of those values, our discussion is focused on highlighting the qualitative behavior of the system rather than providing a specific quantitative extraction of the parameters. From the materials point of view, it is worth noting that U and V are effective interactions projected onto the low energy states, that have a highly non-trivial dependence on the different material parameters of $NbSe_2$. Furthermore, details such as surface effects and substrate effects could impact these parameters.

Finally, we comment on interaction screening effects in $NbSe_2$. From the theoretical point of view, providing an accurate estimate of the screening would require performing RPA (random phase approximation) density functional theory (DFT) calculations of the dielectric screening, which would account for both intraband and interband screening in the system. We note that such estimate cannot be reliably performed with the low energy tight binding model we are considering, as interband contributions would be completely neglected in that scenario.

Estimation of coherence length

To estimate the superconducting coherence length, we first fitted the proximitized spectra of Fig. 4 (c) with Dynes' model. The extracted gap values as function of distance was then plotted with an exponential decay to extract the Coherence length in Fig. S12(a). The fitting formula was $\Delta(x) = \Delta_1 - \Delta_2(1 - e^{\frac{-(x-x_0)}{\xi}})$, where $\Delta(x), \Delta_1, (\Delta_1 - \Delta_2), x_0, \xi$ are fitted gap, SC gap inside the $NbSe_2$ island, residual SC gap in HOPG (due to proximity of nearby islands), spatial location of the boundary between $NbSe_2$ and HOPG and the Coherence length respectively. This fit gives us a Coherence length $\xi \approx 7.3$ nm.

Alternatively, the coherence can be determined from the field dependence of the SC island shown in Fig. S12(b). The SC island size here is 8400 nm^2. From the linear interpolation of the normalized zero bias conductance (ZBC), we can estimate the upper critical field (H_{C2}), which comes out to be ≈ 2.47 T. So, the estimated coherence length will be $\xi = \sqrt{\frac{1}{2\pi}\frac{\phi_0}{H_{c2}}} =$ 11.5 nm.

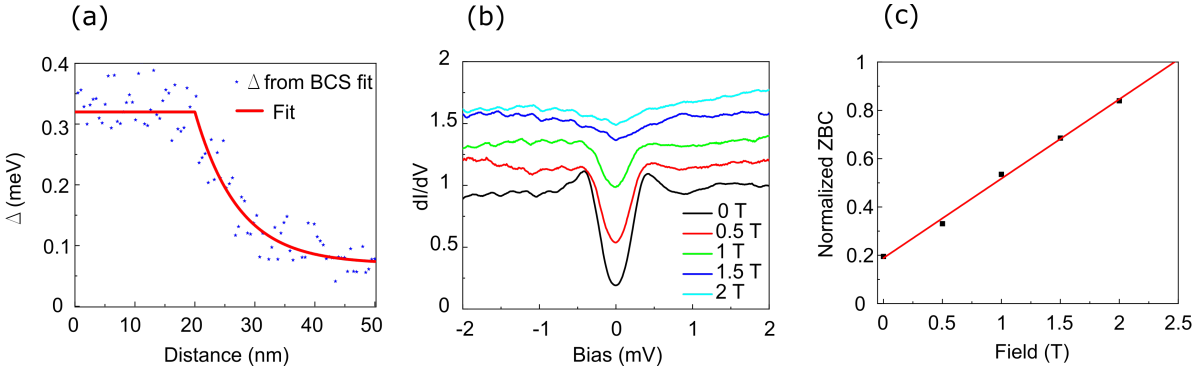

Figure S12: Estimation of Coherence length. (a) Exponential fit of proximitised SC gap. (b) Magnetic field dependence of SC spectra. (c) Linear interpolation of normalized ZBC.

Spatial dependence of islands in proximity

The proximity induced SC gap in the island in Fig. S13(a) varies spatially as illustrated in Fig. S13(b). We observe the presence of SC order by the dip in the conductance at zero bias and the presence of coherence peaks in the different locations of the proximitised island. It indicates that the SC order has been established in the entire island. The SC spectra is asymmetric in conductance values at the coherence peak locations. There is however a variation in the asymmetry observed in the individual spectra at different locations. The histogram of the asymmetry defined by the (normalised conductance at -ve coherence peak location)-(normalised conductance at +ve coherence peak location) shows a distribution asymmetric about zero (mean value of 0.11 from Gaussian fit). The proximitised non-SC island in Fig. S13(d) have variations in the local spectra as seen from Fig. S13(e). Here, the strong electron-hole asymmetries are typical of a non-superconducting origin of the gap,[S6] and support the strongly correlated nature of the small islands.

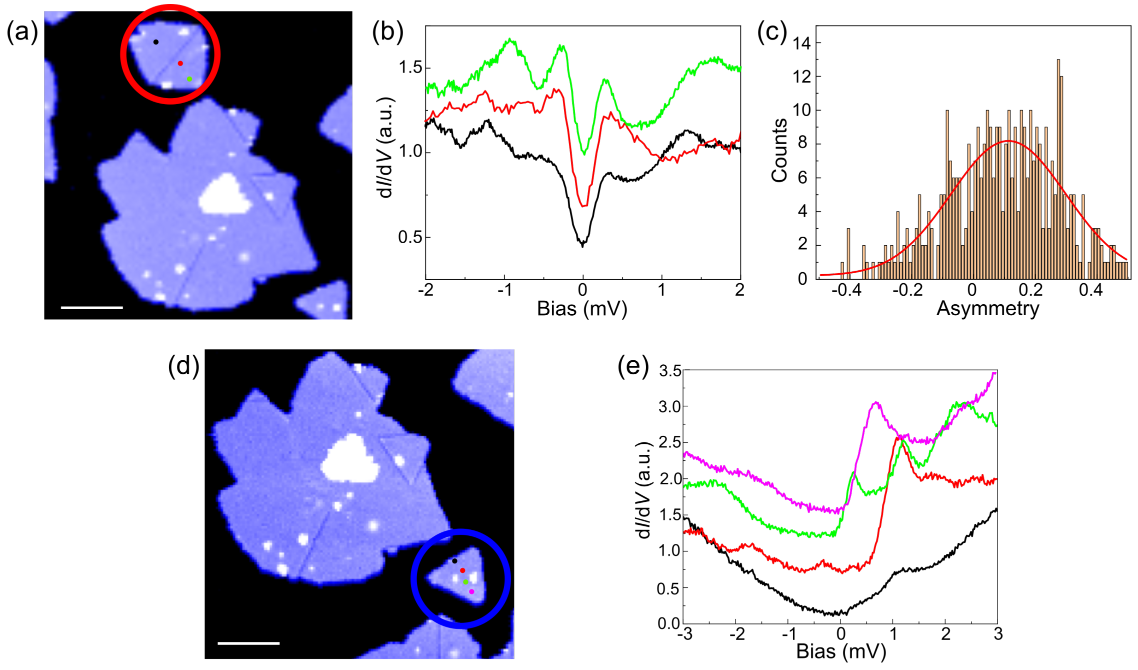

Figure S13: Spatial variation of proximitised spectra. (a) Proximitised superconducting island. Scale bar, 20 nm. (b) Point spectra at the indicated locations. (c) Statistical distribution of the asymmetry in the individual spectra and its Gaussian fit. (d) Proximitised non-superconducting island. Scale bar, 20 nm. (e) Point spectra at the indicated locations.

References

(S1) Horcas, I.; Fernández, R.; Gomez-Rodriguez, J.; Colchero, J.; Gómez-Herrero, J.; Baro, A. WSXM: a software for scanning probe microscopy and a tool for nanotechnology. *Rev. Sci. Instrum.* **2007**, *78*, 013705.

(S2) Yuan, Y.; Wang, X.; Song, C.; Wang, L.; He, K.; Ma, X.; Yao, H.; Li, W.; Xue, Q.-K. Observation of Coulomb Gap and Enhanced Superconducting Gap in Nano-Sized Pb Islands Grown on $SrTiO_3$. *Chin. Phys. Lett.* **2020**, *37*, 017402.

(S3) Ingold, G.-L.; Nazarov, Y. V. In *Single Charge Tunneling: Coulomb Blockade Phenomena In Nanostructures*; Grabert, H., Devoret, M. H., Eds.; Springer: Boston, 1992; pp 21–107.

(S4) Smith, N. V.; Kevan, S. D.; DiSalvo, F. J. Band structures of the layer compounds 1T-TaS_2 and 2H-$TaSe_2$ in the presence of commensurate charge-density waves. *J. Phys. C Solid State Phys.* **1985**, *18*, 3175–3189.

(S5) Kane, C. L.; Mele, E. J. Quantum spin Hall effect in graphene. *Phys. Rev. Lett.* **2005**, *95*, 226801.

(S6) Cai, P.; Ruan, W.; Peng, Y.; Ye, C.; Li, X.; Hao, Z.; Zhou, X.; Lee, D.-H.; Wang, Y. Visualizing the evolution from the Mott insulator to a charge-ordered insulator in lightly doped cuprates. *Nat. Phys.* **2016**, *12*, 1047–1051.
